# Cod otoliths document accelerating climate impacts in the Baltic Sea

**DOI:** 10.1038/s41598-024-67471-2

**Published:** 2024-07-20

**Authors:** Yvette Heimbrand, Karin Limburg, Karin Hüssy, Tomas Næraa, Michele Casini

**Affiliations:** 1 Department of Aquatic Resources, Swedish University of Agricultural Science, Almas Allé 5, Box 7018, 750 07 Uppsala, Sweden; 2 SUNY College of Environmental Science and Forestry, Syracuse, NY 13210 USA; 3 National Institute of Aquatic Resources, Technical University of Denmark, 2800 Kgs. Lyngby, Denmark; 4 Department of Geology, Lund University, Sölvegatan 12, 223 62 Lund, Sweden; 5 Department of Biological, Geological and Environmental Sciences, University of Bologna, Via Selmi 3, 40126 Bologna, Italy

**Keywords:** Ecology, Biogeochemistry, Ecology, Environmental sciences, Ocean sciences

## Abstract

Anthropogenic deoxygenation of the Baltic Sea caused major declines in demersal and benthic habitat quality with consequent impacts on biodiversity and ecosystem services. Using Baltic cod otolith chemical proxies of hypoxia, salinity, and fish metabolic status and growth, we tracked changes from baseline conditions in the late Neolithic (4500 BP) and early twentieth century to the present, in order to understand how recent, accelerating climate change has affected this key species. Otolith hypoxia proxies (Mn:Mg) increased with expanding anoxic water volumes, but decreased with increasing salinity indexed by otolith Sr:Ca. Metabolic status proxied by otolith Mg:Ca and reconstructed growth were positively related to dissolved oxygen percent saturation, with particularly severe declines since 2010. This long-term record of otolith indicators provides further evidence of a profound state change in oxygen for the worse, in one of the world’s largest inland seas. Spreading hypoxia due to climate warming will likely impair fish populations globally and evidence can be tracked with otolith chemical biomarkers.

## Introduction

Growing awareness of deoxygenation in oceans, coastal seas, and inland waters^1,2^ has led to concern about consequences to species’ distributions, population abundances, and biodiversity in general. To address these concerns, efforts have been made to determine biotic baselines under normoxic conditions, whether by spatial comparison^3,4^, temporal trends^5,6^, or both^7,8^. Records of historical and geological hard tissue remains coupled to abiotic information are valuable, but few exist to track hypoxia^9^. However, sclerochronology, the study of temporal physical and chemical variations in the accretion of hard tissues, offers a view on long-term climate patterns^10,11^, and inert, biogenic structures offer insights on global change^5,12–15^.

The Baltic Sea ranks among the best studied large marine ecosystems, with long‐term monitoring time series that serve to document climate and environmental changes^16,17^. This semi-enclosed basin has a history of perennial but intensifying hypoxia and currently contains the largest anthropogenic hypoxic/anoxic region^18^. Narrow, shallow connections to the North Sea limit the inflow of saline, oxygenated water, and ventilation of the stratified bottom layers occurs with diminished frequency and intensity now^19^. Like the Gulf of Mexico and Chesapeake Bay in North America, the northwestern Black Sea, and the Bohai Sea, hypoxia in the Baltic Sea is exacerbated by both agriculture-induced eutrophication^20^ and increased water temperature. Eutrophication stimulates algal blooms that die off, and microbial decomposition consumes oxygen^20^. Warmer water temperature reduces oxygen solubility and increases stratification, thus reducing mixing^1,21^. Although nutrient mitigation has reduced loading to the Baltic during the past 15 years^16,22^, biogeophysical dynamics of the system reintroduce legacy phosphorus from the sediments and maintain the cycle of intense, N-fixing phytoplankton blooms, followed by microbial decomposition and oxygen depletion^18,23,24^.

Against this backdrop, eastern Baltic cod (*Gadus morhua,* hereafter simply referred to as cod), a demersal fish, has emerged as a key study species to examine direct and indirect biological effects of environmental change over time, and the links between hypoxia, habitat impairment, and resulting impacts on its population^6,25^. Increased hypoxia affects metabolic performance negatively as less energy reduces growth and foraging mobility^26,27^. Hypoxia also causes habitat compression, leading to crowding and density-dependent processes, negatively affecting cod body condition^6,28–30^ and reduction of benthic prey for cod^31^. This keystone predator has played an important role in structuring Baltic Sea food webs^32^, and has served as a premier food fish since at least the late Stone Age^33^.

**Figure 1:**
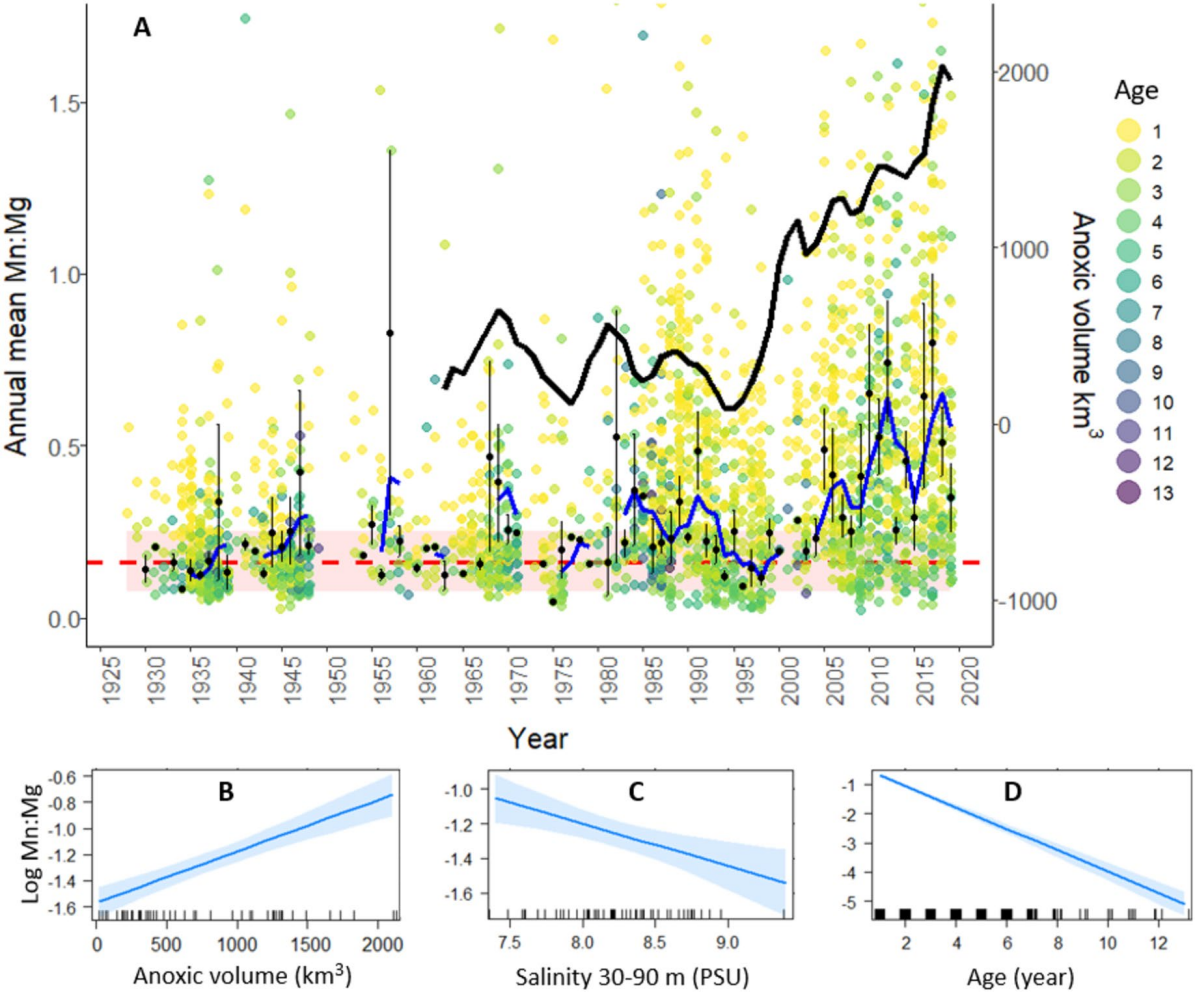
Upper panel: (**A**) Coloured dots represent age specific mean Mn:Mg, used as a chemical proxy for hypoxia exposure for cod (≥ 1 year) in offshore habitats, separated per year within each otolith. The blue line represents the 3-year moving average of the annual mean Mn:Mg for age class 3 (black dots) ± se (vertical lines). The black line represents the 3-year moving average of annual anoxic (≤ 0 ml/l O_2_) volume (km^3^) from 1960 to 2019 in the Baltic Sea. Normoxic baseline illustrated by mean Mn:Mg from Neolithic samples (dashed red horizontal line) and standard deviation (pink band). Lower panel: Repeated measurements mixed model’s significant fixed effects of (**B**) anoxic volume (km^3^), (**C**) salinity (PSU) at 30–90 m depth in the Gotland Deep (station BY15, quarter 4) and (**D**) age (years) on otolith log transformed mean Mn:Mg per year.

During the twentieth century, increased nutrient loading drove up primary production and had an initially positive effect on cod recruitment from the 1940s to 1980s^34^. Cod spawning biomass peaked in the “boom years” of the late 1970s-early 1980s, setting the century record, in response also to reduced fishing pressure and uniquely favourable hydrographic conditions^35^. This was followed by a major decline caused by a combination of overfishing and deteriorating environmental conditions^34^, including increase in hypoxic areas (Fig. 1). Reduced maximum length, growth, condition, size at maturation and reproductive potential were all signs of a population in distress^25^ and by the 2010s the eastern Baltic cod population had plunged to critically low levels that led to an emergency closing of the fishery^36,37^.

Otoliths are calcified structures that form part of the fish auditory system and grow throughout life, depositing increments formed of aragonite on a scaffolding of proteins^38^, with annual and daily periodicities making them particularly useful in age and growth studies^39^. Understanding direct hypoxia impacts on Baltic cod has become possible through the use of chemical proxies deposited in their otoliths. Limburg, et al.^5^ first identified lifetime patterns of the trace element manganese as a potential proxy for Baltic cod hypoxia exposure, as the dissolved forms, Mn^2+^ and Mn^3+^, are available under low redox conditions^40–42^. Additionally, otolith magnesium (as Mg:Ca ratios) correlates with growth and metabolism^43^ as well as fish condition^30^. Strontium:calcium (Sr:Ca) ratios can often be used as salinity proxies^44^, and have been useful to track cod movements^45^.

Further studies revealed: (a) a close correspondence of otolith hypoxia proxies to oceanographic estimates of water < 2 ml/l oxygen^29^, (b) that fish with low body condition were exposed to hypoxia^30^, and (c) that average cod condition declined with increased hypoxia exposure^28,30^.

Declining condition of Baltic cod also led to a worsening of contrast in annual increment patterns in their otoliths^46^, such that the age-based stock assessment was severely hampered^47^. Newer, non-traditional ageing methods using otolith chemistry have reduced uncertainty in age estimation^43,48,49^. These take advantage of seasonal variations in metabolically sensitive trace elements as well as those which vary from external, seasonally influenced biogeochemistry^48^. These advances enable comparisons of contemporary Baltic cod, whose health is currently severely compromised, with cod from earlier periods.

We use a rare collection of Baltic cod otoliths extending from the late Neolithic (ca 5000–4500 BP) and the past century to examine individual cod life histories and hypoxia exposure^5,29^. Although episodes of hypoxia and anoxia in the Baltic extend back thousands of years^50^, our Neolithic site was shallow and normoxic 5000–4500 BP^5,50^, based on sediment cores^50^. These provide a normoxic baseline against which we can compare modern Baltic cod. We can therefore use otolith chemistry to (1) document long-term trends in hypoxia exposure by cod; (2) assess hypoxia exposure in juvenile and adult habitats by reconstructing salinity; (3) track individual metabolic status; and (4) monitor decadal changes in cod growth rate. We present evidence that environmental changes led to a worsened situation for Baltic cod.

## Results and discussion

### General hypoxia exposure patterns

We use cod otolith chemistry as an observational tool to document the long-term worsening environmental conditions experienced by cod within the demersal habitats these fish occupy. Neolithic Mn:Mg levels provided a normoxic baseline below mean values observed at any time during the modern era, although closest to the period before 1980.

Cod (≥ Age 1) otolith annual mean Mn:Mg, our growth adjusted proxy of hypoxia exposure, varied within years and was elevated throughout modern samples compared to the Neolithic baseline (Fig. 1A). However beginning around 1980 Mn:Mg increased noticeably, dropping in the early 1990s and then increasing dramatically after that. The time-series of anoxic volume followed the same pattern with a sharp increase from the late 1990s (Fig. 1A). A linear mixed-effects model for repeated measures (model formula: log annual mean Mn:Mg ~ anoxic volume + salinity + age + (age | fish ID) + (1 | year)) predicting the otolith hypoxia proxy from the fixed effects (anoxia, salinity, and age) was highly statistically significant (conditional R^2^ = 0.82; fixed effects marginal R^2^ = 0.39; *P* < 0.001) with no multicollinearity; Fig. 1 shows the positive effect of anoxia and negative effect of salinity and age on Mn:Mg.

### The ontogenetic aspect of hypoxia exposure

**Figure 2:**
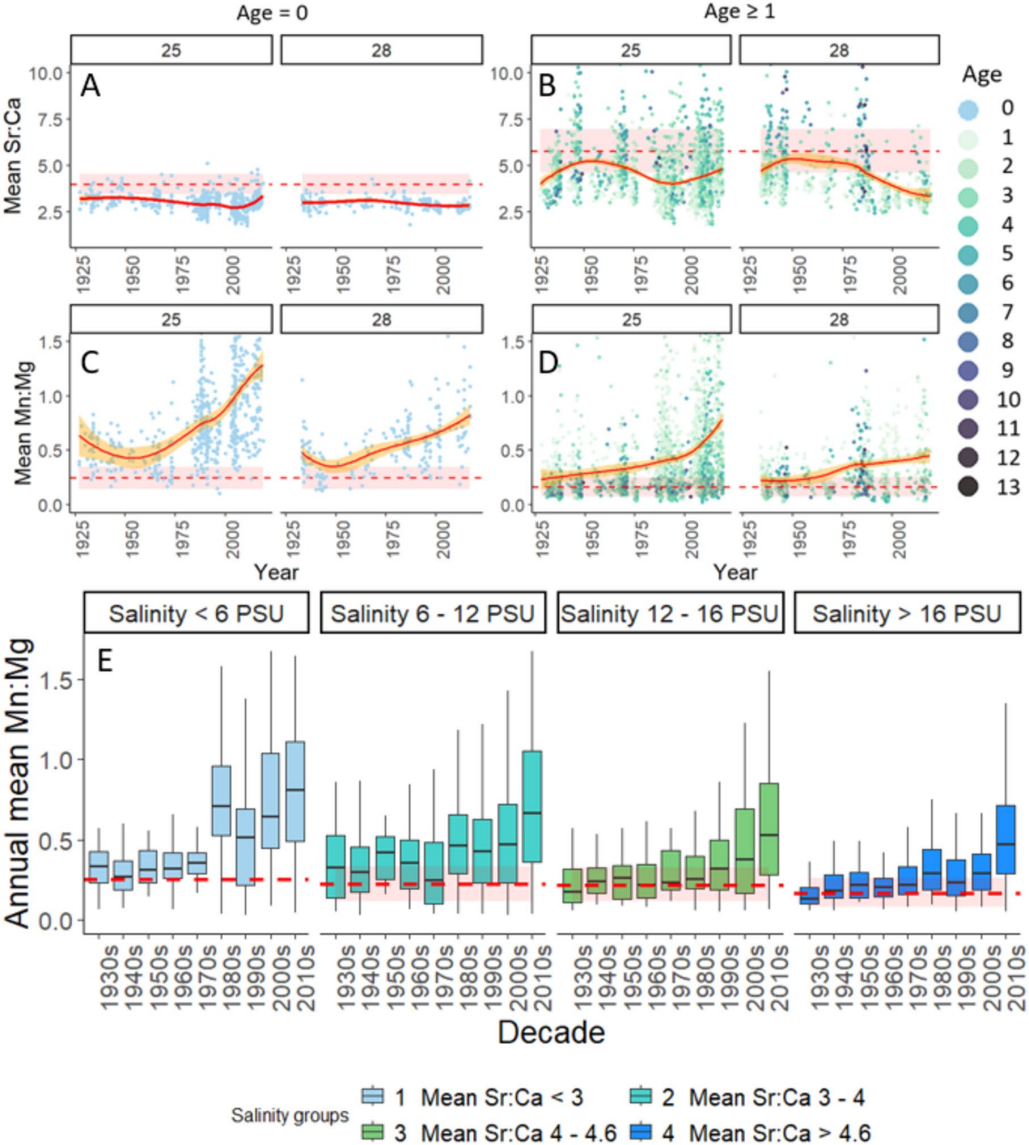
Salinity (**A**,**B**) and hypoxia (**C**,**D**) proxies from cod otoliths for Age-0 fish (left) and Ages ≥ 1 (right) for ICES Subdivisions 25 and 28. Red splines indicate mean value per year with 95% confidence interval in orange. Corresponding mean Neolithic baselines by age group are shown as red dashed horizontal lines and standard deviation (sd) as pink bands around them. Coloured dots represent age specific mean Mn:Mg, separated per year within each otolith. (**E**) Growth-adjusted hypoxia exposures (mean within year Mn:Mg) for all age classes by decades per salinity group based on Sr:Ca ratios. Neolithic baselines by salinity group are shown as red dashed horizontal lines ± sd. Note that there are not enough data points for calculation of sd for salinity < 6 PSU (salinity group 1) for the Neolithic time period. Box = 25th and 75th percentiles; horizontal line = median value; whiskers = 1.5 times interquartile range above 75th percentile or below 25th percentile.

In the last 115 years, hypoxia in the open Baltic Sea has increased tenfold, mainly linked to eutrophication^17^ and this is evidenced in cod otoliths (Fig. 2). Hypoxia exposure indexed by mean annual Mn:Mg ratios varied widely within years, but patterns across time emerged. We separated chemical signals corresponding to the first year of life (Age 0) from older fish because the former use inshore, lower salinity habitats^51^, here proxied by Sr:Ca (Fig. 2 A, top left panel, mean annual Sr:Ca 95% CI [2.64, 2.68]). These areas are generally shallower and thus thought to be less prone to hypoxia exposure. However, there is evidence that coastal hypoxia has increased steadily since the mid-1970s^52^. Our study showed that young cod (Fig. 2C, middle left panel) recorded at least as much if not more hypoxia exposure as older fish with worsening conditions since the 1970s (Fig. 2D, middle right panel). This may have led to recruitment bottlenecks and in part may have contributed to overall loss of resilience in the population.

For both age groups, hypoxia indices rose more slowly in ICES SD 28 (International Council for the Exploration of the Sea subdivision, northeastern Baltic Proper, see “Methods” and Fig. 5D for explanation of SD) compared to SD 25 (southwestern Baltic proper, Fig. 2). Hypoxia exposure was low until 1960, increased gradually through the late 1990s, and accelerated in the 2000s (Fig. 2). Compared to the Neolithic baselines (Fig. 2, dashed horizontal lines), Age-0 modern indices were elevated, and for Ages ≥ 1, despite much scatter, the trends exceeded the Neolithic baseline. Decadal increases in hypoxia were significant in both SDs, with notable increases after 1980 (Kruskal–Wallis tests and Wilcoxon tests, Table S4–S11). Hypoxia exposure was generally greater at lower salinities (Fig. 2E, bottom left panel). Mn:Mg increased over time in all salinity groups beginning around the 1980s and was especially pronounced in the lowest salinity group, corresponding to coastal juvenile nursing grounds (Fig. 2, top left panel). Pairwise comparison of decades within each salinity group showed that cod generally experienced significantly greater hypoxic exposure in the 2010s compared to other decades (Fig. 2E, lower panel; Kruskal–Wallis tests and Wilcoxon tests, Tables S12–S15).

The freshwater inputs from precipitation and river discharge of the Baltic Sea catchment area, minus evaporation, generally regulate multidecadal variations in mean salinity. However, major Baltic inflows from the North Sea have a greater effect on the salinity stratification on a timescale of > 4 years^53^. In the 1980s, a hypoxic era occurred, followed by a well-oxygenated period in the early 1990s, but then both hypoxia and anoxia expanded and intensified to the highest observed levels during the 2010s. Annual values of otolith Mn:Mg, our hypoxia proxy, showed significant, positive correlation to time series of anoxic volumes. We posit that increased dissolved Mn became available for uptake, due to redox conditions linked to exacerbated deoxygenation over time. Mn:Mg decreased with increasing salinity, indicating that inflows from the North Sea, temporarily renewing the Baltic with saline, oxygenated water, reduced hypoxia exposure^19^. Nevertheless, increasing hypoxia exposure from the 1980s was evident in otoliths from cod caught in both juvenile and adult habitats in different salinities. Due to the strong vertical and horizontal salinity gradients in the Baltic Sea^54^, further analyses are needed to examine whether these results indicate hypoxia at depth or are due to migration. Taken together, our results suggest that eastern Baltic cod find it increasingly more difficult to avoid exposure to hypoxia as it expands in the Baltic Sea.

### Metabolic status

**Figure 3:**
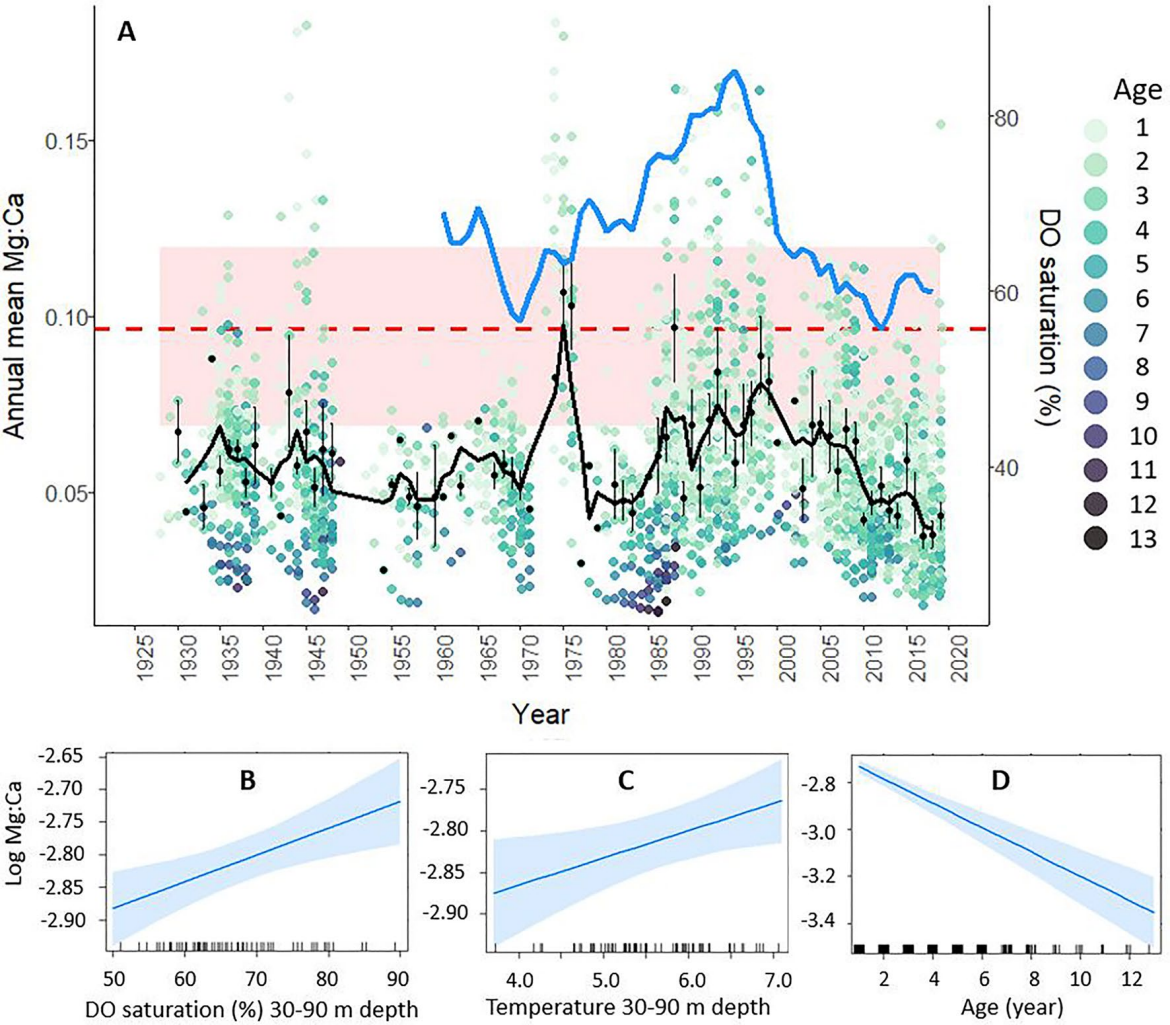
Upper panel (**A**) Coloured dots in a gradient from pale green to dark blue-grey, represent age-specific mean Mg:Ca, used as a chemical proxy for metabolic activity for cod (≥ 1 year) in offshore habitats, separated per year within each otolith. The black line represents the 3-year moving average of the annual mean Mg:Ca for age class 3 (black dots) ± se (vertical lines). The blue line represents the 3-year moving average of dissolved oxygen percent saturation (DO) from 1960–2020 at 30–90 m depth in the Gotland Deep (station BY15). Neolithic baseline for mean Mg:Ca is illustrated by the dashed red horizontal line ± sd (pink horizontal band). Lower panel: Repeated measures mixed model’s significant fixed effects of (**B**) dissolved oxygen percent saturation (DO) at 30–90 m depth in the Gotland Deep (station BY15), (**C**) temperature at 30–90 m depth in the Gotland Deep (station BY15 quarter 4 and (**D**) age on otolith log transformed mean Mg:Ca per year.

Metabolic status indexed by log transformed mean Mg:Ca per calendar year allowed us to study changes in cod metabolic activity in relation to environmental changes (mean temperature at 30–90 m depth (October–December) and mean dissolved oxygen percent saturation at 30–90 m depth in the Gotland Deep, Station BY15) on an annual level (Fig. 3). Annual mean Mg:Ca ratios declined with fish age (and size), consistent with specific metabolic rates^55^; the Neolithic ratios exceeded all modern observations (Table S17). To avoid chemical influences from ontogenetic and environmental properties connected to juvenile habitats, age class 0 was excluded to focus on older cod in offshore habitats. The overall trend in Mg:Ca indicates fluctuations from the 1930s to the 1970s. From the 1980s, mean Mg:Ca increased with highest levels toward the end of the 1990s, followed by a decline during the 2000s and 2010s (Fig. 3). The linear mixed-effects model for repeated measures (model formula: log annual mean Mg:Ca ~ oxygen percent saturation + temperature + age + (age | fish ID) + (1 | year)) found the model's total explanatory power for predicting log Mg:Ca substantial (conditional R^2^ 0.90, fixed effects marginal R^2^ 0.06) with no multicollinearity. The effect of annual mean dissolved oxygen percent saturation and temperature was statistically significant and positive (Fig. 3, *P* < 0.05), whereas the effect of age was statistically significant and negative (Fig. 3, *P* < 0.001).

The temporal trend in otolith Mg:Ca, our proxy for metabolism and growth^43^, coincides very well with the temporal development of cod condition and growth shown in previous studies, including the rapid decline after the early 1990s^6,56^. This rapid decline in cod condition and growth^6,57^ coincides with increasing exposure to hypoxia (our study), indicating a direct effect of oxygen worsening^28,30^. The metabolic proxy Mg:Ca in our study was also positively related to the mean temperature at 30–90 m depth in the Gotland Deep in quarter 4 (Fig. 3C), which ranged between 3.7 and 7.1 °C. These results correspond well with field studies presenting direct evidence of a positive relation between otolith Mg and temperature in data storage tagged wild adult cod in the southern Baltic Sea^58^ as well as laboratory experiments of Atlantic cod showing that the optimal temperature for growth of adult cod was 7 °C^59^. However, one might expect a negative effect on cod metabolism if warming continues within hypoxic habitat, since that would further reduce oxygen solubility and exacerbate hypoxia^21,60^. Deepwater increases in temperature and salinity in the central Baltic result from hydrographic transport from the Danish Straits through other basins in the south^18^. As this water ages, it becomes hypoxic and, increasingly, anoxic^61^.

The metabolic status reflected by mean Mg:Ca appears to have been high during the normoxic Neolithic (Fig. 3, Table S7), and relatively stable levels occurred from the 1930s to the 1960s. The extraordinarily high Mg:Ca values in 1972–1976 might be related to an overall increase in ecosystem productivity around that time driven by nutrient loading and internal recycling^62^, resulting in major increases in secondary production due to favourable environmental conditions^63^. Growth rates in young cod would therefore have accelerated, along with Mg:Ca. Increased levels during the 1980s and 1990s, especially in SD 25, reflect times of documented high growth rates^56^, whereafter Mg:Ca levels fluctuate and decline (Fig. 3).

### Growth patterns over time

**Figure 4:**
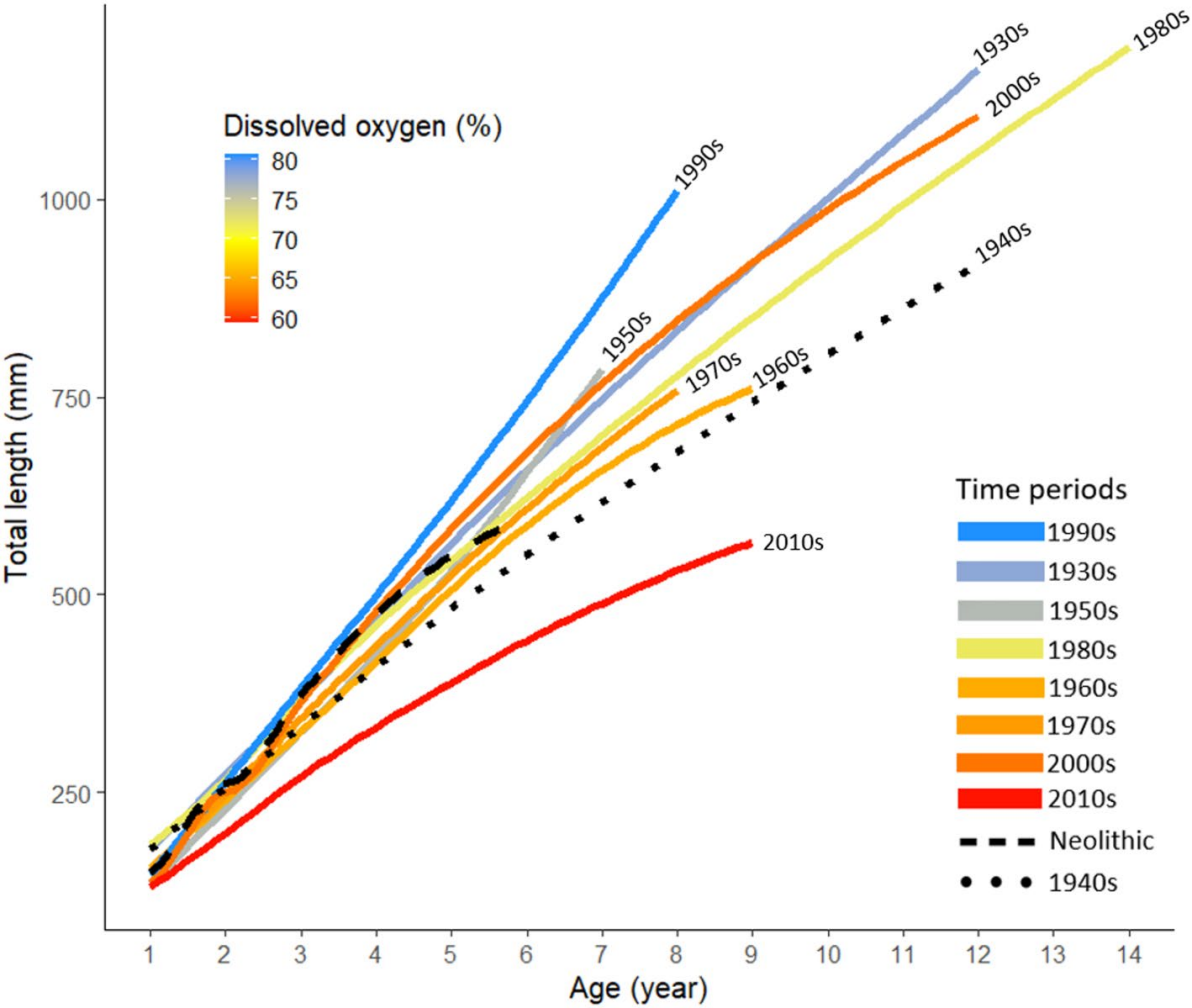
Predicted mean length at age for eastern Baltic cod over time in the Baltic Sea, based on chemically back-calculated length at age for each year of a fish’s life. Decades are colour coded to display the corresponding mean dissolved oxygen saturation per decade. There were no dissolved oxygen percent saturation data available for 1940s (black dotted line) and the Neolithic time period (dashed black line) at 30–90 m depth in the Gotland Deep (station BY15).

**Figure 5:**
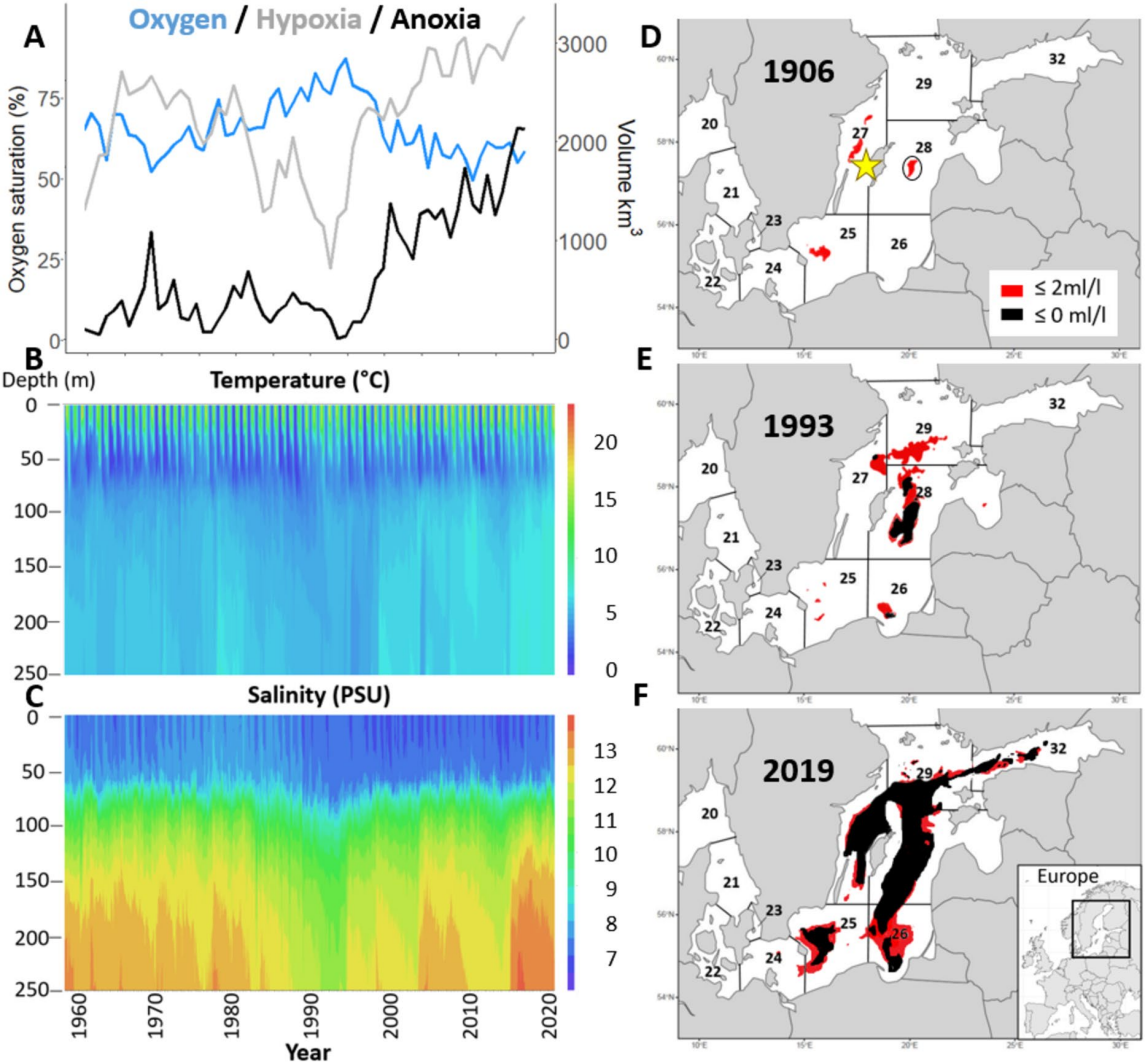
Abiotic environmental data over time in the Baltic Sea. (**A**) 3-year moving average of dissolved oxygen (DO) percent saturation (blue) measured at 30–90 m depth at monitoring station BY15 in the Gotland Deep, calculated according to^83^ from annual mean oxygen (ml/l). Annual anoxic (≤ 0 ml/l O_2_, black) and hypoxic (< 2 ml/l O_2_,grey) volumes (km^3^) in the Baltic Sea retrieved from Hansson and Viktorsson^84^. Quarterly data on temperature (**B**) and salinity (**C**) from station BY15. DO, temperature, and salinity data were compiled with the Nest system from the Baltic Nest Institute’s Baltic Environmental Data base (BED), http://nest.su.se and included data provided by the Swedish Meteorological and Hydrological Institute (SMHI), https://sharkweb.smhi.se/). (**D**–**F**) Areal extent of hypoxia/anoxia in 1906, 1993 and 2019, respectively per ICES SD (20–29 and 32) in the Baltic Sea. Neolithic site is indicated by a star and the Gotland Deep (station BY15), by a black a ring in panel (**D**). Maps (**D**–**F**) were generated with R Studio software version 4.1.2^85^ (package sf, rnaturalearthdata, rnaturalearth, sp, ggplot2 and wk) and modified from Carstensen, et al.^17^ and Hansson and Viktorsson^84^ in PowerPoint.

A repeated measures analysis, using chemically back-calculated length at age for each year of a fish’s life (model formula: length ~ age, random =  ~ 1| fish ID), predicted greatest growth rate and mean length at age in the 1990s, the decade with the highest mean dissolved oxygen saturation when hypoxia was at a 60-year low^23^ (Fig. 4). Conversely, the model predicted a substantial decrease in mean length at age, which clearly separates from the other growth curves by age 2, in the hypoxia-intensified 2010s (Fig. 4, Table S16). Comparing size at Age 7, a cod in the 1990s would average 875 mm and in the 2010s, only 488 mm—a 56% decrease in size at comparable age. There were no dissolved oxygen percent saturation data available for 1940s and the Neolithic time period. Comparing the growth curve for the Neolithic time period with modern cod indicated similar length at age 6 as in the 1970s and 1980s (Fig. 4). The mean oxygen saturation at the monitoring station in the northeastern Baltic Proper (SD 28, Fig. 5D) was the second lowest of all decades for the 2000s. However, large inflows from the North Sea in 2003 and 2004^19^ likely affected cod in the southern Baltic positively, contributing to the good growth in the 2000s (Fig. 4).

With climate change driving the combined effect of deoxygenation and ocean warming, a reduction in fish body size is expected^64–66^. This appears to be the case for eastern Baltic cod (Fig. 4). The markedly low length-at-age growth curve for the 2010s and corresponding declines in body condition^28^ could suggest a critical tipping point for a population collapse triggered by hypoxia. In a statistical analysis of factors affecting maximum total length of Baltic cod, hypoxic exposure had a significantly greater effect than either fishing pressure or prey availability^67^.

Increased anoxic bottom areas can affect cod condition and growth also indirectly, since it causes loss of benthos and may affect even the hypoxia tolerant preferred benthic prey *Saduria entomon*^68^, if anoxia is persistent. Lindmark, et al.^69^ observed a positive effect of *Saduria entomon* biomass density on cod condition. Dietary analyses of cod caught 1963–1988 showed that the mass of *Saduria entomon* in cod stomachs increased with increasing cod size, but declined in all sizes of cod caught 1994–2014^70^.

However, apart hypoxia, a complex of other factors are likely involved in worsened cod condition and growth. A rebound in the seal population increased cod predation and intensified parasite transmission^71,72^. Habitat compression due to hypoxia has led to reduced distribution and increased spatial overlap with the competing flounder (*Platichthys flesus* and *Platichthys solemdali*)^6,73,74^. At the same time, the abundances of clupeid fish, important food for larger cod, have declined in the central Baltic^6,75^. Together, warming, hypoxia, changes in the food web and historic size selective overfishing^37,76^ have triggered a syndrome of interactions resulting in smaller, less healthy cod^6^.

Otolith Mg:Ca for age classes ≥ 1 year correlated positively with dissolved oxygen percent saturation over time (Fig. 3B), further indicating that today’s severe hypoxic exposure entails consequences such as metabolic stress, substantially affecting cod growth and condition negatively.

### Pre-historic baselines

The use of archaeological archives, such as otoliths for paleoenvironmental reconstruction relies on the preservation of the chemical composition, which may be altered during post-depositional diagenetic processes^77,78^. Heating and cooking might also change the chemical composition in pre-historic otoliths, but with less alteration for elements likely to substitute for Ca^2+^^79,80^. Therefore, we do not expect the divalent positive ions (Sr^2+^, Mg^2+^ and Mn^2+^) in the Neolithic samples used to create a historic baseline in this study to be affected. Two dimensional mapping to visualize trace elemental content in otoliths from archaeological sites has improved data reliability and interpretation of diagenesis^81^ (Fig. S1), enabling reconstruction of past habitat utilization and migration patterns^82^.

### Otoliths as bioindicators for environmental and biological changes

Long-term climate change is increasingly revealed through bioindicators^11^. Technological advances in data loggers and other water sampling methods in the sea can provide a wide range of detailed measurements of environmental conditions. However, they are unable to measure directly individual fishes’ response to climate change and biological consequences for fish populations. The combination of otolith chronometric chemical properties and the fact that fisheries agencies around the world annually collect otoliths from many fish species from a wide variety of habitats potentially provides an invaluable asset of diverse bioindicators, covering a substantial span in time and space. This information, offering both temporal and spatial resolution of climate change, is important for management advice, protection of fish populations, and setting policies and implementing measures for reducing hypoxia in impacted systems such as the Baltic Sea.

Climate and anthropogenic drivers that affect aquatic systems leave “geochemical footprints”, some of which can be interpreted with fish otolith microchemical tracers. Fish otoliths can warn of declining environmental conditions, and, moreover, track hypoxia and enable reconstruction of historical marine environmental changes. Eastern Baltic cod otoliths from the Swedish Neolithic era and the last century were examined to explore the accelerating climate impacts in the Baltic Sea causing exposure to hypoxia, metabolic stress and biological changes in the fish. When an otolith is analysed across its entire span, the chemical patterns reveal both exogenous processes (biogeochemistry, physical drivers) and physiology. Taken together, this detailed information for every individual fish provides powerful insights that scale up to populations and ecosystems. Otolith chemical proxies offer a promising framework for future laboratory and field studies of other taxa, including sessile invertebrates, linking trace elemental incorporation in biominerals to environmental drivers, in order to forecast future impacts.

## Methods

### Ethics statement

No live fish were used in this specific study. From archives, we used collections of fish ear stones (otoliths) which are small biominerals of calcium carbonate. The samples were originally collected by other research projects from the 1930s to the 2010s. Part of the fish from these projects were purchased from commercial fishermen, while part were fished during scientific research expeditions for fisheries management purposes. With new emerging techniques we could reuse the otoliths in the archives for a new purpose, to assess how environmental stressors have affected Baltic cod over time. The fish surveys that have been conducted during the last century have not always been regarded as animal experimentation according to the Swedish legislation. During the 1990s the Swedish Board of Agriculture initiated discussions with the Swedish Board of Fisheries regarding these issues and those discussions ended with a statement that fish caught during fish surveys should not be regarded as experimental animals. However, this changed over the following decades and since 2003 the number of fishes caught at fish surveys have been reported to the Swedish Board of Agriculture, despite the fact the fact that the legal aspects had not been clarified. Since 2012 the marine fish surveys have had permissions from regional ethical boards (permit numbers 255-2012, 126-2015 and 5.8.18-06684/2020). Sweden is part of the International Council for the Exploration of the Sea (ICES), which is an intergovernmental organization coordinating marine research and developing unbiased, non-political scientific advice for fisheries management. ICES was established in 1902 and received a legal foundation and full international status through an agreed convention in 1964.

### Sample collection

Modern cod otoliths were selected from available samples of cod caught from the 1930s through the 2010s in both commercial catches and scientific surveys in the International Council for the Exploration of the Sea (ICES) subdivisions (SD) 25 (n = 601) and 28 (n = 198) of the Baltic Sea (Fig. 5, shown in panels D–F). These otoliths were part of collections by the Department of Aquatic Resources at the Swedish University of Agricultural Sciences and the former Swedish Board of Fisheries stored at the Swedish National Archives. Additionally, we chose 46 Neolithic specimens recovered from an archaeological site on the island of Gotland in ICES SD 27^33^ (Fig. 5). Samples represent four generalized periods: (1) the Neolithic as a prehistoric baseline; (2) the 1930s–1970s as a modern period of low hypoxia and fairly oligotrophic conditions; (3) the 1980s representing moderate hypoxia with strong cod year-classes, followed by low hypoxia and strong cod growth in the 1990s; and (4) the 2000s, with variable water inflows from the North Sea and increasing hypoxia, particularly after 2010. Where possible, up to 5 samples per 5 cm length class were randomly selected per decade from both commercial catches and scientific surveys. In total, we included otoliths from 845 cod in this study (Table S1).

### Hydrographic data

The central Baltic’s Gotland Deep (Station BY15, Fig. 5) was selected to represent large scale, long-term changes due to the completeness of the data. Long-term records of water temperature, dissolved oxygen (DO), and salinity were obtained from the Baltic Nest Institute’s Baltic Environmental Database (BED; http://nest.su.se) that included data provided by the Swedish Meteorological and Hydrological Institute (SMHI; https://sharkweb.smhi.se/). documented that the depth distribution of cod in the Baltic Sea has increased over time, focusing on quarter 4 (October–December). To cover the general habitat depth range for cod, we calculated the mean salinity and temperature at 30–90 m depth in quarter 4. Annual mean DO (ml/l) at 30–90 m depth was converted to percent saturation^83^. Areal (km^2^) and volumetric (km^3^) extents of hypoxia (< 2 ml/l O_2_) and anoxia (< 0 ml/l O_2_) have been estimated by SMHI since 1960 and are available as reports^84^. We tested percent saturation, temperature, salinity and volumetric anoxia extents in relation to our otolith chemical proxies of hypoxia exposure and metabolic status.

### Otolith analysis

We analysed otolith trace elemental concentrations with laser ablation inductively coupled plasma mass spectrometry (LA-ICP-MS) at the Department of Geology at Lund University in Lund, Sweden (n = 407) and at the College of Environmental Science and Forestry at the State University of New York (SUNY-ESF) in Syracuse, New York USA (n = 438). Detailed instrumental settings and otolith preparation methods are described in Tables S2 and S3 and in Heimbrand et al.^48^.

Sample analyses were done in sequences with reference material NIST SRM 610, NIST SRM 612^86^ and MACS-3^87^, with a standard-sample-standard bracketing setup. Analyses were run as line scans that were pre-ablated prior to analyses. At Lund University, NIST SRM 610 and 612 were analysed to check daily performance and MACS-3 was used as the primary standard for quantifying the results. The ESF facility used NIST SRM 612 for daily performance and MACS-3 and MAPS-4^88,89^ as primary standards. Tuning of the instruments was done using NIST SRM 612 with emphasis on having stable signal counts on relevant isotopes, and low oxide production (i.e. below 0.5%). Helium was used as the carrier gas and Ar was added downstream of the sample chamber. The laser was set up to run with a spot size of 90 × 56 μm (Lund University) and 35–110 µm diameter round spot (ESF Facility), a fluence of 2 J/cm^2^ on the carbonate and 3 J/cm^2^ on the NIST glasses, a scanning speed of 24 μm/s and a repetition rate of 16 Hz (Lund University), and 3–7 μm/s and a repetition rate of 10 Hz at ESF.

Data reduction and statistical analyses for trace elements (ppm) were performed in Excel and Iolite^90,91^. The trace elements included in this study were strontium (measured as ^88^Sr), magnesium (^24,25,26^ Mg), manganese (^55^Mn) and phosphorus (^31^P), all in ratio to calcium (^43^Ca). Polyatomic interference of ^48^Ca^2+^ on ^24^Mg^+^^92^ was discovered when the same set of samples were analysed at both facilities for quality control. The interference resulted in constant overestimation of quantified Mg concentrations measured by the LA-ICP-MS in SUNY-ESF, which were corrected by multiplying values by 0.6 to enable comparisons with the results from the LA-ICP-MS in Lund.

Many of the Neolithic otoliths displayed evidence of partial diagenesis, with visible cracks that contained high Mn (Fig. S1). Most could be analysed on their dorsal axes, deleting the Mn highly elevated outliers as needed (> 3 standard deviations). For the few Neolithic otoliths that had excessive diagenetic contamination on the dorsal axis, data from the proximal axis were used instead, and distance was normalized to the dorsal axis.

All samples were chemically aged by interpreting seasonal minima and maxima in concentration of otolith Mg:Ca and P:Ca, following Heimbrand et al.^48^ and Hüssy et al.^49^. Trace elemental ratios were thereafter parsed per calendar year as mean values within annual growth zones.

### Statistical analyses

Statistical analyses were performed using R Studio software version 4.1.2^85^ with α < 0.05 as the threshold for statistical significance. Graphical comparisons were made with the R package ggplot2^93,94^. The otolith chemistry data (see next section) consisted of repeated measurements on each otolith. We used this trace element data in ratio to calcium as a response variable to conduct mixed-effects regression analyses (lmer) with the R package lme4^95^. To assess statistical power of the linear mixed-effects models for repeated measures within this study and ensure that the predictor variables were not highly correlated, the variance inflation factor (VIF) was tested to check multicollinearity. Predictor variables were considered highly correlated if VIF > 5, which was not the case for any of the models within this study. When the assumption for normality and homoscedasticity was not met, a nonparametric Kruskal–Wallis and pairwise Wilcoxon tests with Bonferroni adjusted p-values were selected for comparisons of different time periods and habitats. More statistical details are given for each model below and in the R code, available at the GitHub repository https://github.com/YveHei/Baltic-cod.

### Proxies to assess hypoxia exposure and salinity

Annual mean Mn:Mg values were calculated for every year of all individual fishes’ life and used as a proxy for hypoxia exposure. Dividing Mn by Mg adjusts Mn for the influence of growth on its uptake^30^. Mn:Mg was not normally distributed and therefore transformed logarithmically. A repeated measures linear mixed-effect model (lmer) was fitted (estimated using REML and the nloptwrap optimizer)^95^ for the time period 1960–2019 to assess the effect of environmental and biological factors on log transformed otolith Mn:Mg over time for cod (≥ 1 year). The annual anoxic volume in the Baltic Sea (km^3^) and the annual mean salinity at 30–90 m depth in the Gotland Deep (station BY15 in quarter 4 (October–December)) were selected as fixed factors to represent large scale, long-term environmental changes linked to hypoxia. To account for biological lifetime changes in the otolith uptake of Mn:Mg, age was also included as a fixed factor. Individual fish ID nested within age and calendar year were used as random factors (model formula: log annual mean Mn:Mg ~ anoxic volume + salinity + age + (age | fish ID) + (1 | year)). The Wald t-distribution approximation was used for computing *P*-values. Measurements corresponding to juveniles (age = 0 years old), were not included in the model as they are known to reside in shallower coastal habitat^96^. In order to assess differences in hypoxia exposure in juvenile and adult cod habitats, annual mean Mn:Mg values were divided into two age groups. The first year of life (by convention Age 0), representing juvenile cod in shallow, coastal habitats (“nurseries”)^97,98^, and Age ≥ 1 year, older cod in offshore, deeper habitats^28^.

Habitat salinities correlate to different levels of Sr:Ca in cod otoliths^45,51^. Sr:Ca proxies of salinity were broken into 4 groups to represent low to high salinity based on annual mean Sr:Ca: (1) < 3.0, (2) 3.0–4.0, (3) 4.0–4.6 and (4) > 4.6. The salinity (PSU) was estimated from otolith Sr (ppm), using the natural log–log linear Sr-salinity relation from a calibration curve^51^ with the equation:

$$Predicted salinity=exp(\left(\mathit{ln}N(Sr\right)-6.241)/0.442$$

The calibration curve was based on otolith Sr from cod in the Kattegat Sea, North Sea, eastern and western Baltic and salinity at catch^45^. For simplicity, salinity is here approximately estimated per group: (1) < 6 PSU, (2) 6–12 PSU, (3) 12–16 PSU and (4) > 16 PSU.

The assumption for normality and homoscedasticity was not met for otolith mean Mn:Mg parsed per calendar year. Therefore, nonparametric Kruskal–Wallis and pairwise Wilcoxon tests with Bonferroni adjusted p-values were selected for comparisons of decadal hypoxic exposure dependent on salinity. As there were few data points representing the 1950s (N = 3) and the Neolithic time period (N = 1) in salinity group 1, these were not included in the statistical comparisons with other decades.

### Proxy to assess metabolic status

Chemical age estimation allowed for extracting annual mean Mg:Ca, computed to proxy metabolism/growth per calendar year^43,48^. Hence, the number of repeated measurements per otolith corresponded to the age of the fish. The proxy, Mg:Ca did not meet the requirements of normal distribution and was log transformed. We fitted a repeated measures linear mixed-effect model (estimated using REML and nloptwrap optimizer) for the time period 1960–2019 to assess the effect of environmental and biological factors on log transformed otolith Mg:Ca. The annual mean dissolved oxygen percent saturation at 30–90 m depth in the Gotland Deep (BY15) and the mean temperature at 30–90 m depth in quarter 4 (October–December) were selected as fixed factors to represent large scale, long-term environmental changes linked to fish metabolism. To account for biological lifetime changes in the otolith uptake of Mg:Ca, age was also included as a fixed factor. Individual fish ID nested within age and calendar year were used as random effects (model formula: log annual mean Mg:Ca ~ oxygen percent saturation + temperature + age + (age | fish ID) + (1 | year)). The Wald t-distribution approximation was used for computing p-values. Measurements corresponding to juveniles (age = 0 years old), were not included in the model as they are known to reside in a shallower coastal habitat^96^.

### Length and growth estimation

For samples lacking information about total length (Neolithic samples), a linear regression based on the relationship between total length and the distance from the otolith core to the dorsal edge of cod within this study was used to back calculate the total length with the equation:

$$\begin{gathered} Total\,length \, \left( {mm} \right)\, = \,0.1323 \, \times \, dorsal\,otolith\,distance \, \left( {\mu m} \right) - 31.033 \hfill \\ \left( {ANOVA, \, df\, = \,1,707,{R^2}\, = \,0.83,p\, < \,0.05} \right). \hfill \\ \end{gathered}$$

Retrospective lengths-at-age were back-calculated from otolith increments by means of the equation^99^:

$$Back{ - }calculated \, length \, at \, age \, \left( {mm} \right) \, = \, TL \, + \, \left( {{O_a}- \, {O_c}} \right) \, \times \, \left( {TL \, - \, {L_i}} \right) \, / \, \left( {{O_c} - \, {O_i}} \right)$$

where *TL* is the Total length of the fish at capture (mm). *O*_*a*_ is the annulus distance from the otolith core (µm). *O*_*c*_ is the distance from the otolith core to the dorsal edge at capture (µm). *L*_*i*_ is the length of the fish at the biological intercept (4 mm), i.e., the total length of a newly hatched cod larvae^100^. *O*_*i*_ is the otolith radius at the biological intercept (hatch) 10 µm^100^.

We performed a repeated measures ANOVA to predict mean fish length at age per decade and the Neolithic time period with chemically estimated length at age as the response variable, age as the fixed effect, and individual fish as a random effect (model formula: length ~ age, random =  ~ 1| fish ID).

### Supplementary Information

Supplementary Information.

## Data Availability

The datasets generated and analysed within this study and R code are available at the GitHub repository https://github.com/YveHei/Baltic-cod.
