# Supplementary material for: Cod otoliths document accelerating climate impacts in the Baltic Sea
Source: Sci Rep. 2024 Jul 20;14:16750. doi: 10.1038/s41598-024-67471-2 (PMC11271452; doi:10.1038/s41598-024-67471-2)
Supplement: Supplementary file 1 — Supplementary Information. [file 41598_2024_67471_MOESM1_ESM.pdf]

Supplementary information

Cod Otoliths Document Accelerating Climate Impacts in the Baltic Sea

Heimbrand, Y., Limburg, K., Hüssy, K., Næraa, T. & Casini, M.

Table S1. Overview of sample collection of eastern Baltic cod per time period, ICES SD and length group.

5 cm length class groups	1930s		1940s		1950s		1960s		1970s		1980s		1990s		2000s		2010s		Neolithic 4500-5000 YBP
ICES SD	25	28	25	28	25	28	25	28	25	28	25	28	25	28	25	28	25	28	27
0-5															7				
6-10											2				11				
11-15											1				1				
16-20			4								2	2	1	2	14	3	34	1	
21-25	1		3			1	1				24	2	4	4	12	4	10		1
26-30	1		2				2		2		18	3	9	2	6	3	12	3	2
31-35	3	2	2	1		3	5	3			9	3	9	3	2	3	16	4	6
36-40	2	4	3	3		1	3	7	5		4	2	16	2	7	3	34	7	5
41-45	3	3	2	3	3		3	4	2		4	2	18	2	15	3	35	6	7
46-50	2	5	3	1	1	1	2	2	7		5	3	12	3	11	3	15	6	11
51-55	3	6	2	3			3		5		3	4	10	2	5			1	10
56-60	3	3	3	3			2		5		2	3	11	5	3	1	3		2
61-65	4	2	2	4			2		1		3	2	7	2	2	1			1
66-70	2	3	3	1	1		2		1		3	5	6	1	4				
71-75	3	3	1	4	1		1	1			3	3	3		2				
76-80	2		1								2	2	3		5				1
81-85	2						1				1	2	4		2				
86-90	3		1									1			2				
91-95	1						1					1	1						
96-100											1		1						
101-105	1																		
106-110												1							
N=845	36	31	32	23	6	6	28	17	28	0	87	41	115	28	111	23	159	28	46

Table S2. Setup for the laser ablation inductively coupled plasma mass spectrometry (LA-ICP-MS) at the Department of Geology at Lund University in Lund, Sweden.

**Laser ablation system**	**Lund University**
Make, Model & type	Photon machines, Analyte G2 excimer laser
Ablation cell & volume	HelEx 2-volume cell with eQC
Laser wavelength	193 nm
Pulse width	<4 ns
Fluence	2 J/cm^2 on otolith and MACS-3 and 3 J/cm^2 on NIST glass
Repetition rate	16 Hz
Scan speed	24 µm/s
Spot size	56 x 90 µm
Background collection	30 seconds prior to each analysis
Ablation setup	Line scan; 30 seconds on standards
Cell carrier gas flow	0.8 l/min He
ICP-MS Instrument	
Make, Model & type	Bruker Aurora Elite Quadrupole ICP-MS
RF power	Ca. 1300 W
Make-up gas flow	Ca. 0.95 l/min Ar
Detection system	Single collector discrete dynode electron multiplier or DDEM
Masses measured (dwell time in milliseconds)	^{24}Mg(12), ^{25}Mg(12), ^{26}Mg(12), ^{31}P(13), ^{43}Ca(10), ^{55}Mn(12), ^{63}Cu(16),^{66}Zn(16), ^{88}Sr(8), ^{137}Ba(10), ^{138}Ba(10)
**Data reduction**	
Software	Iolite v3.63
Primary standard	NIST SRM 610 - preferred values from Jochum et al. 2011. OR MACS-3 Prefered values from GeoReM (8/2019)
Secondary standard(s)	NIST SRM 612
Internal standard	^{43}Ca - Ca mean concentration in otolith set to 38 wt.%

Table S3. Setup for the laser ablation inductively coupled plasma mass spectrometry (LA-ICP-MS) at the College of Environmental Science and Forestry at the State University of New York (SUNY-ESF) in Syracuse, New York USA.

**Facility**	**College of Environmental Sciences and Forestry at the State University of New York, Syracuse, NY, USA**
Instrument	Single collector laser ablation inductively coupled plasma mass spectrometry (SC-LA-ICP-MS)
Laser	193-nm solid state New Wave (ESI) UP laser
Spot shape for line transect	Circular
Spot size for line transect (µm)	35-110 diameter
Laser scan speed (µm/s)	3 - 7
Data reduction software	Excel
Carrier, makeup-, auxiliary- and cooling gas	Helium
Average laser power (%)	90
Repetition rate (Hz)	10
Standardization for otolith calcium carbonate matrix	carbonate pellet MACS3 and MAPS-4
Validation tool	glass NIST-612 standard

Table S4. Post hoc pairwise comparisons using Wilcoxon rank sum test with Bonferroni adjusted p-values of annual mean Mn:Mg per decade for age class = 0 in SD25. Kruskal-Wallis chi-squared = 213.25, df = 8, p-value < $2.2e^{-16}$.

	1930s	1940s	1950s	1960s	1970s	1980s	1990s	2000s
1940s	1.00000	-	-	-	-	-	-	-
1950s	1.00000	1.00000	-	-	-	-	-	-
1960s	1.00000	1.00000	1.00000	-	-	-	-	-
1970s	0.04407	1.00000	1.00000	1.00000	-	-	-	-
1980s	0.00436	$3.6e^{-06}$	0.28916	0.00012	$1.7e^{-06}$	-	-	-
1990s	0.10249	$1.7e^{-05}$	0.41328	0.00100	$6.9e^{-06}$	1.00000	-	-
2000s	$3.8e^{-06}$	$5.3e^{-10}$	0.04067	$1.1e^{-07}$	$1.4e^{-09}$	0.24496	0.00025	-
2010s	$2.8e^{-11}$	$5.3e^{-14}$	0.00504	$5.3e^{-11}$	$2.5e^{-12}$	$4.9e^{-08}$	$1.4e^{-14}$	0.04561

Table S5. Post hoc pairwise comparisons using Wilcoxon rank sum test with Bonferroni adjusted p-values of annual mean Mn:Mg per decade for age class > 0 in SD25. Kruskal-Wallis chi-squared = 303.97, df = 8, p-value $< 2.2e^{-16}$.

	1930s	1940s	1950s	1960s	1970s	1980s	1990s	2000s
1940s	0.12736	-	-	-	-	-	-	-
1950s	0.15693	1.00000	-	-	-	-	-	-
1960s	0.04029	1.00000	1.00000	-	-	-	-	-
1970s	0.33933	1.00000	1.00000	1.00000	-	-	-	-
1980s	$2.7e^{-13}$	$3.0e^{-05}$	1.00000	$3.6e^{-05}$	0.00051	-	-	-
1990s	$1.3e^{-10}$	0.01557	1.00000	0.02125	0.05814	1.00000	-	-
2000s	$2.3e^{-13}$	$9.3e^{-05}$	1.00000	0.00020	0.00190	1.00000	1.00000	-
2010s	$< 2.0e^{-16}$	$<2.0e^{-16}$	0.00368	$< 2.0e^{-16}$	$< 2.0e^{-16}$	$1.5e^{-07}$	$< 2.0e^{-16}$	$6.9e^{-11}$

Table S6. Post hoc pairwise comparisons using Wilcoxon rank sum test with Bonferroni adjusted p-values of annual mean Mn:Mg per decade for age class = 0 in SD28. Kruskal-Wallis chi-squared = 80.625, df = 7, p-value $= 1.027e^{-14}$.

	1930s	1940s	1950s	1960s	1980s	1990s	2000s
1940s	0.00065	-	-	-	-	-	-
1950s	1.00000	1.00000	-	-	-	-	-
1960s	1.00000	0.23102	1.00000	-	-	-	-
1980s	0.00463	$9.1e^{-10}$	0.13266	0.00091	-	-	-
1990s	1.00000	0.00030	1.00000	1.00000	0.02089	-	-
2000s	0.00686	$5.0e^{-07}$	0.11610	0.00221	1.00000	0.01496	-
2010s	0.00019	$1.2e^{-08}$	0.02640	0.00010	0.43784	0.00042	1.00000

Table S7. Post hoc pairwise comparisons using Wilcoxon rank sum test with Bonferroni adjusted p-values of annual mean Mn:Mg per decade for age class > 0 in SD28. Kruskal-Wallis chi-squared = 112.57, df = 7, p-value $< 2.2e^{-16}$.

	1930s	1940s	1950s	1960s	1980s	1990s	2000s
1940s	0.71386	-	-	-	-	-	-
1950s	0.42189	1.00000	-	-	-	-	-
1960s	1.00000	1.00000	1.00000	-	-	-	-
1980s	$2.9e^{-13}$	$1.9e^{-08}$	1.00000	0.00065	-	-	-
1990s	1.00000	1.00000	1.00000	1.00000	0.00494	-	-
2000s	$5.5e^{-10}$	$2.5e^{-07}$	0.16671	$8.1e^{-05}$	1.00000	0.00153	-
2010s	$2.6e^{-09}$	$5.5e^{-06}$	1.00000	0.00318	1.00000	0.00805	1.00000

Table S8. Post hoc pairwise comparisons using Wilcoxon rank sum test with Bonferroni adjusted p-values of annual mean Sr:Ca per decade for age class = 0 in SD25. Kruskal-Wallis chi-squared = 91.551, df = 8, p-value = $2.251e^{-16}$.

	1930s	1940s	1950s	1960s	1970s	1980s	1990s	2000s
1940s	1.0000	-	-	-	-	-	-	-
1950s	1.0000	1.0000	-	-	-	-	-	-
1960s	1.0000	1.0000	1.0000	-	-	-	-	-
1970s	1.0000	1.0000	1.0000	1.0000	-	-	-	-
1980s	$3.2e^{-06}$	$9.9e^{-06}$	0.0939	0.0131	0.0174	-	-	-
1990s	0.0152	0.0354	1.0000	1.0000	1.0000	1.0000	-	-
2000s	$3.9e^{-08}$	$4.0e^{-08}$	0.0651	$6.9e^{-05}$	$4.9e^{-05}$	0.1393	0.0042	-
2010s	0.0079	0.0223	0.8797	1.0000	1.0000	0.2450	1.0000	$2.0e^{-05}$

Table S9. Post hoc pairwise comparisons using Wilcoxon rank sum test with Bonferroni adjusted p-values of annual mean Sr:Ca per decade for age class > 0 in SD25. Kruskal-Wallis chi-squared = 153.61, df = 8, p-value < $2.2e^{-16}$.

	1930s	1940s	1950s	1960s	1970s	1980s	1990s	2000s
1940s	$3.4e^{-10}$	-	-	-	-	-	-	-
1950s	1.0000	0.0256	-	-	-	-	-	-
1960s	0.0043	0.2077	1.0000	-	-	-	-	-
1970s	1.0000	0.0004	1.0000	1.0000	-	-	-	-
1980s	0.3202	$9.1e^{-13}$	1.0000	$2.9e^{-06}$	0.0096	-	-	-
1990s	$5.5e^{-05}$	$< 2.0e^{-16}$	0.1812	$3.4e^{-11}$	$1.3e^{-06}$	1.0000	-	-
2000s	0.4370	$7.2e^{-14}$	1.0000	$2.8e^{-06}$	0.0064	1.0000	1.0000	-
2010s	1.0000	$7.6e^{-10}$	1.0000	0.0159	1.0000	0.2498	$3.7e^{-07}$	0.1086

Table S10. Post hoc pairwise comparisons using Wilcoxon rank sum test with Bonferroni adjusted p-values of annual mean Sr:Ca per decade for age class = 0 in SD28. Kruskal-Wallis chi-squared = 28.786, df = 7, p-value = 0.0001583.

	1930s	1940s	1950s	1960s	1980s	1990s	2000s
1940s	1.00000	-	-	-	-	-	-
1950s	1.00000	1.00000	-	-	-	-	-
1960s	1.00000	1.00000	1.00000	-	-	-	-
1980s	1.00000	1.00000	1.00000	1.00000	-	-	-
1990s	1.00000	1.00000	1.00000	1.00000	1.00000	-	-
2000s	0.00435	0.15306	0.03659	0.00704	0.14882	0.00061	-
2010s	0.27456	1.00000	0.71698	0.34740	1.00000	0.08736	1.00000

Table S11. Post hoc pairwise comparisons using Wilcoxon rank sum test with Bonferroni adjusted p-values of annual mean Sr:Ca per decade for age class > 0 in SD28. Kruskal-Wallis chi-squared = 217.63, df = 7, p-value < $2.2e^{-16}$.

	1930s	1940s	1950s	1960s	1980s	1990s	2000s
1940s	0.810	-	-	-	-	-	-
1950s	1.000	0.903	-	-	-	-	-
1960s	1.000	1.000	1.000	-	-	-	-
1980s	1.000	0.014	1.000	1.000	-	-	-
1990s	$< 2.0e^{-16}$	$< 2.0e^{-16}$	$4.4e^{-05}$	$1.9e^{-12}$	$2.9e^{-12}$	-	-
2000s	$< 2.0e^{-16}$	$< 2.0e^{-16}$	$1.3e^{-07}$	$5.2e^{-12}$	$1.1e^{-12}$	0.392	-
2010s	$1.3e^{-12}$	$8.9e^{-15}$	0.024	$2.1e^{-08}$	$2.6e^{-08}$	1.000	0.032

Table S12. Post hoc pairwise comparisons using Wilcoxon rank sum test of annual mean Mn:Mg per decade and salinity group 1, with Bonferroni adjusted p-values. In Salinity group 1 there were few data points representing the 1950s (N=3) and the Neolithic time period (N=1), hence these were exclude from statistical analyses. Kruskal-Wallis chi-squared = 139.83, df = 7, p-value < $2.2e\text{-}^{16}$.

Salinity group 1

	1930s	1940s	1960s	1970s	1980s	1990s	2000s
1940s	1.0000	-	-	-	-	-	-
1960s	1.0000	1.0000	-	-	-	-	-
1970s	1.0000	1.0000	1.0000	-	-	-	-
1980s	1.5e-07	5.2e-09	3.8e-05	0.0096	-	-	-
1990s	0.1534	0.0164	1.0000	1.0000	1.3e-05	-	-
2000s	6.3e-08	6.5e-10	3.8e-05	0.0062	1.0000	1.5e-06	-
2010s	1.6e-07	6.8e-09	5.3e-05	0.0089	0.6649	3.9e-10	1.000

Table S13. Post hoc pairwise comparisons using Wilcoxon rank sum test of annual mean Mn:Mg per decade and salinity group 2, with Bonferroni adjusted p-values. Kruskal-Wallis chi-squared = 169.87, df = 9, p-value < $2.2e\text{-}^{16}$.

Salinity group 2

	1930s	1940s	1950s	1960s	1970s	1980s	1990s	2000s	2010s
1940s	1.00000	-	-	-	-	-	-	-	-
1950s	1.00000	1.00000	-	-	-	-	-	-	-
1960s	1.00000	1.00000	1.00000	-	-	-	-	-	-
1970s	1.00000	1.00000	1.00000	1.00000	-	-	-	-	-
1980s	0.00025	0.00521	1.00000	0.22075	0.00188	-	-	-	-
1990s	0.05784	0.31714	1.00000	1.00000	0.04644	1.00000	-	-	-
2000s	0.00213	0.02467	1.00000	0.62780	0.00723	1.00000	1.00000	-	-
2010s	$< 2.0e^{-16}$	$1.4e^{-10}$	0.08735	$1.5e^{-07}$	$4.4e^{-09}$	$6.9e^{-08}$	$1.1e^{-11}$	$5.1e^{-05}$	-
Neolithic	0.52806	0.63315	0.00448	0.04731	1.00000	$3.2e^{-06}$	0.00045	0.00014	$1.4e^{-09}$

Table S14. Post hoc pairwise comparisons using Wilcoxon rank sum test of annual mean Mn:Mg per decade and salinity group 3, with Bonferroni adjusted p-values Kruskal-Wallis chi-squared = 80.743, df = 9, p-value = 1.151e-^{13}.

Salinity group 3

	1930s	1940s	1950s	1960s	1970s	1980s	1990s	2000s	2010s
1940s	1.00000	-	-	-	-	-	-	-	-
1950s	1.00000	1.00000	-	-	-	-	-	-	-
1960s	1.00000	1.00000	1.00000	-	-	-	-	-	-
1970s	1.00000	1.00000	1.00000	1.00000	-	-	-	-	-
1980s	0.05015	1.00000	1.00000	1.00000	1.00000	-	-	-	-
1990s	0.01518	1.00000	1.00000	1.00000	1.00000	1.00000	-	-	-
2000s	0.10922	1.00000	1.00000	1.00000	1.00000	1.00000	1.00000	-	-
2010s	$1.2e^{-10}$	$1.7e^{-06}$	0.20990	$5.8e^{-05}$	0.03066	0.00017	0.00068	0.73363	-
Neolithic	1.00000	1.00000	1.00000	1.00000	1.00000	0.54124	0.21048	0.86365	$2.2e^{-06}$

Table S15. Post hoc pairwise comparisons using Wilcoxon rank sum test of annual mean Mn:Mg per decade and salinity group 4, with Bonferroni adjusted p-values. Kruskal-Wallis chi-squared = 292.59, df = 9, p-value < 2.2e-^{16}.

Salinity group 4

	1930s	1940s	1950s	1960s	1970s	1980s	1990s	2000s	2010s
1940s	0.00106	-	-	-	-	-	-	-	-
1950s	0.41213	1.00000	-	-	-	-	-	-	-
1960s	0.00153	1.00000	1.00000	-	-	-	-	-	-
1970s	0.00012	1.00000	1.00000	1.00000	-	-	-	-	-
1980s	$3.5e^{-14}$	$5.0e^{-05}$	0.86549	0.00061	1.00000	-	-	-	-
1990s	$3.8e^{-07}$	0.51028	1.00000	1.00000	1.00000	1.00000	-	-	-
2000s	$2.3e^{-10}$	0.00118	1.00000	0.00324	1.00000	1.00000	1.00000	-	-
2010s	$< 2.0e^{-16}$	$< 2.0e^{-16}$	0.00062	$4.7e^{-16}$	$9.6e^{-07}$	$2.3e^{-07}$	$1.4e^{-08}$	$1.8e^{-06}$	-
Neolithic	1.00000	0.00244	0.87874	0.00762	0.00016	$4.3e^{-14}$	$8.5e^{-07}$	$1.5e^{-10}$	$< 2.0e^{-16}$

Table S16. Predicted mean length at age per time period, based on annotated annuli distance from otolith core on the dorsal side of the transverse cross section.

Eastern Baltic cod	Predicted mean length (mm) at age ± standard deviation													
Time period	1	2	3	4	5	6	7	8	9	10	11	12	13	14
Neolithic (n=46)	148	261	373	483	543	599	NA	NA	NA	NA	NA	NA	NA	NA
	±57	±57	±59	±70	±56	±18								
1930s (n=67)	178	273	368	465	565	660	744	826	915	981	1075	1189	NA	NA
	±45	±45	±46	±48	±51	±55	±46	±62	±68	±78	±78	±99		
1940s (n=55)	178	255	333	407	483	552	604	685	752	780	855	930	NA	NA
	±62	±63	±66	±68	±67	±69	±67	±73	±97	±137	±137	±137		
1950s	134	230	326	422	527	681	766	NA	NA	NA	NA	NA	NA	NA
(n=12)	±46	±46	±50	±50	±68	±163	NA							
1960s	154	240	327	415	508	617	665	706	763	NA	NA	NA	NA	NA
(n=43)	±58	±58	±61	±74	±84	±60	±125	±153	±172					
1970s	151	247	344	436	523	594	669	768	NA	NA	NA	NA	NA	NA
(n=28)	±52	±52	±52	±54	±44	±107	±41	NA						
1980s (n=128)	183	267	368	465	539	612	700	781	845	927	959	1055	1138	1218
	±52	±52	±62	±64	±65	±71	±78	±82	±90	±117	±31	±18	±18	±NA
1990s	146	263	382	499	615	742	875	1034	NA	NA	NA	NA	NA	NA
(n=144)	±52	±52	±58	±63	±71	±79	±76	NA						
2000s (n=135)	135	250	364	480	590	670	778	770	876	950	NA	NA	NA	NA
	±61	±70	±79	±91	±99	±94	±112	±46	±46	NA				
2010s (n=187)	130	198	270	333	387	446	488	512	589	NA	NA	NA	NA	NA
	±44	±44	±49	±50	±51	±56	±69	±26	NA					

Table S17. Age specific mean values for Mg:Ca within year for all samples in the study and age specific threshold for hypoxia measured as mean Mn:Mg within year according to [29].

Age class	Mean Mg:Ca within year for moderns samples (1930s – 2010s)	Mean Mg:Ca within year for Neolithic samples	Threshold for hypoxia (Mean Mn:Mg within year)
1	0.0687	0.0998	0.0532
2	0.0682	0.0911	0.0400
3	0.0662	0.0925	0.0214
4	0.0604	0.0921	0.0134
5	0.0560	0.0878	0.0090
6	0.0449	0.1139	0.0084
7	0.0364		0.0084
8	0.0338		0.0084
9	0.0324		0.0084
10	0.0276		0.0084
11	0.0290		0.0084
12	0.0265		0.0084
13	0.0251		0.0084
14	0.0192		0.0084

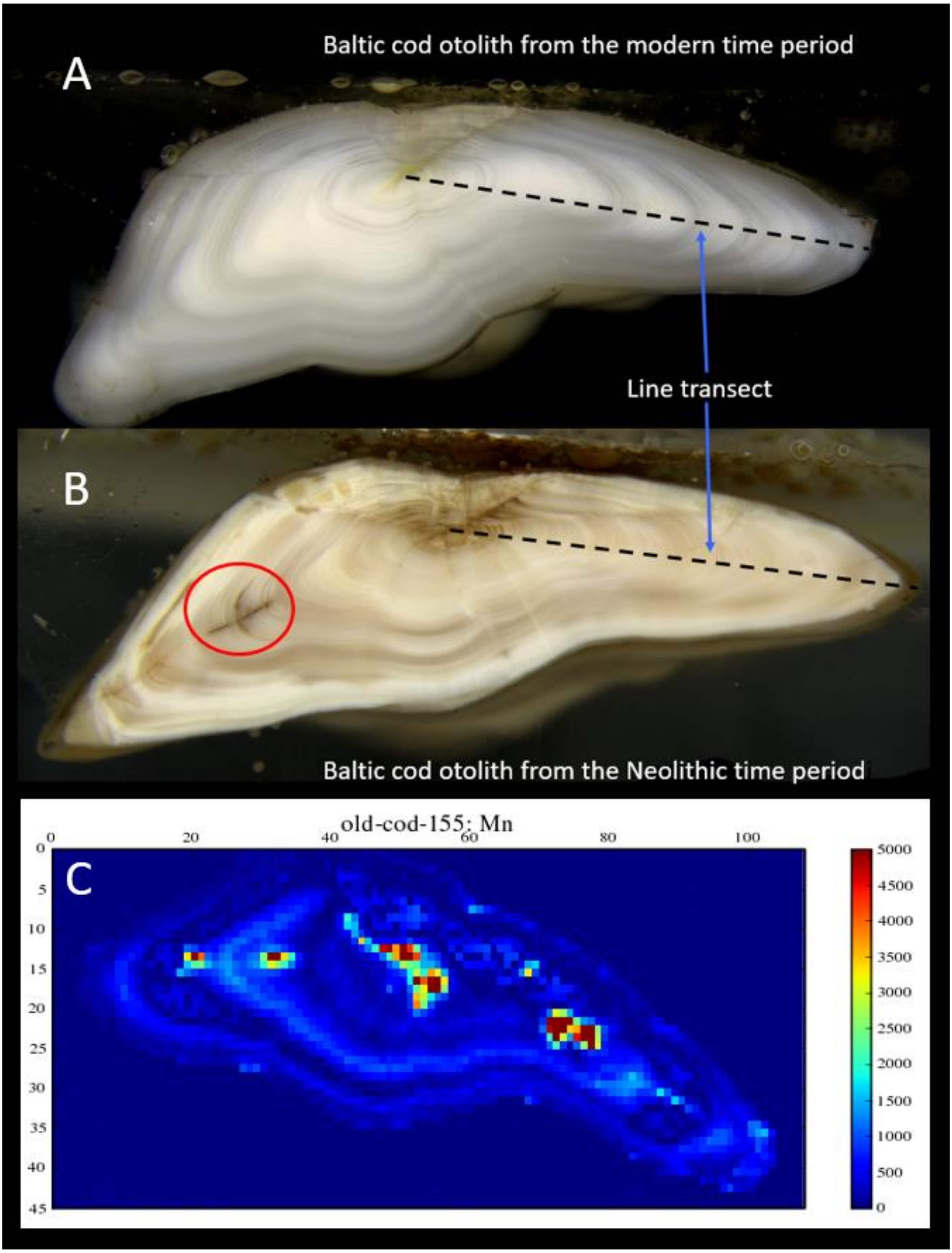

Figure.S1. A: Photo of a Baltic cod otolith from the modern time period. B: Photo of a Neolithic cod otolith from the Baltic Sea with visible cracks (red circle). C: Two dimensional mapping of the manganese (Mn) concentration in another Neolithic cod otolith from the Baltic Sea displaying partial diagenesis, with visible cracks (green to red pixels) containing high Mn levels. For the few Neolithic otoliths that had excessive diagenetic contamination on the dorsal axis, data from the proximal axis were used instead, and distance was normalized to the dorsal axis as in panel C.
